# Supplementary figures and images for: Genome-wide association meta-analysis identifies pleiotropic risk loci for aerodigestive squamous cell cancers
Source: PLoS Genet. 2021 Mar 5;17(3):e1009254. doi: 10.1371/journal.pgen.1009254 (PMC7968735; doi:10.1371/journal.pgen.1009254)

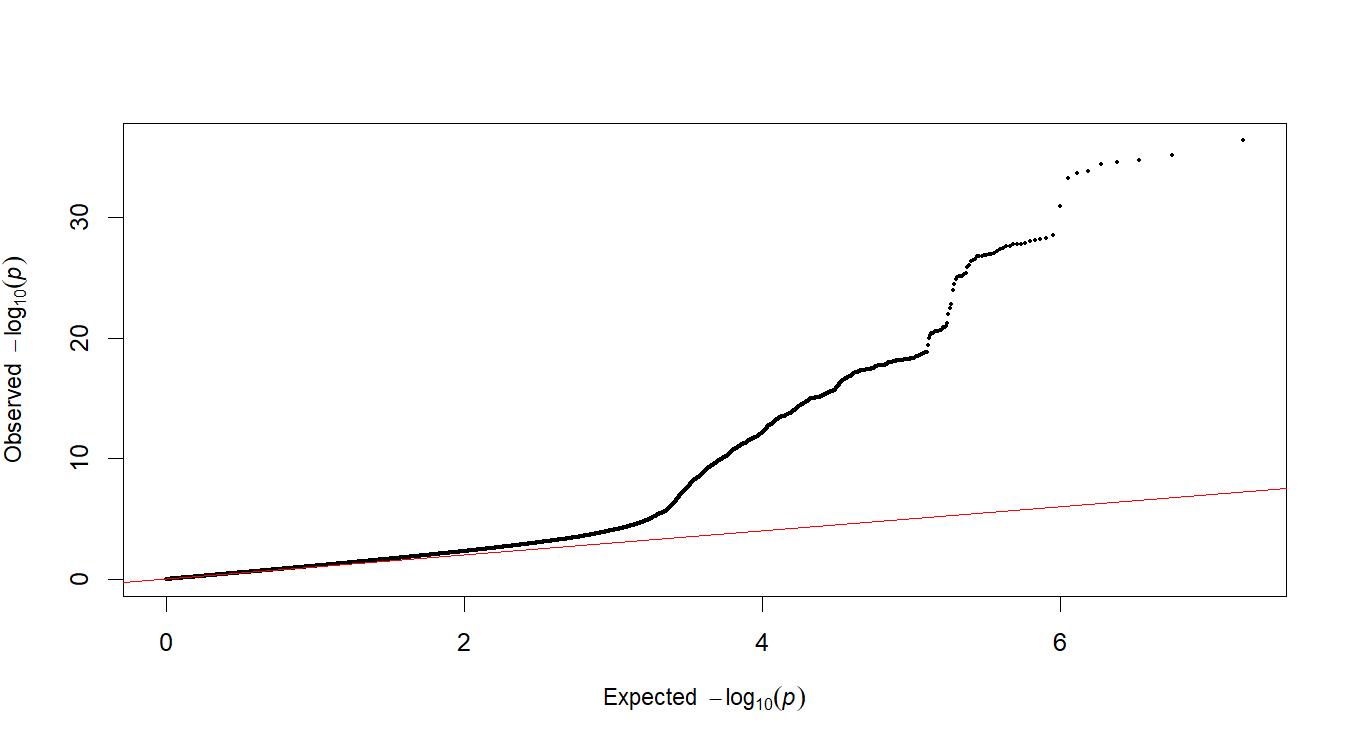

Supplement: S1 Fig — Quantile-quantile plot of the p-values for ASSET F-E meta-analyses results including lung, oral/oropharyngeal, larynx and esophageal SqCCs. (corrected λ = 1.006). (TIFF) [file pgen.1009254.s001.tiff]

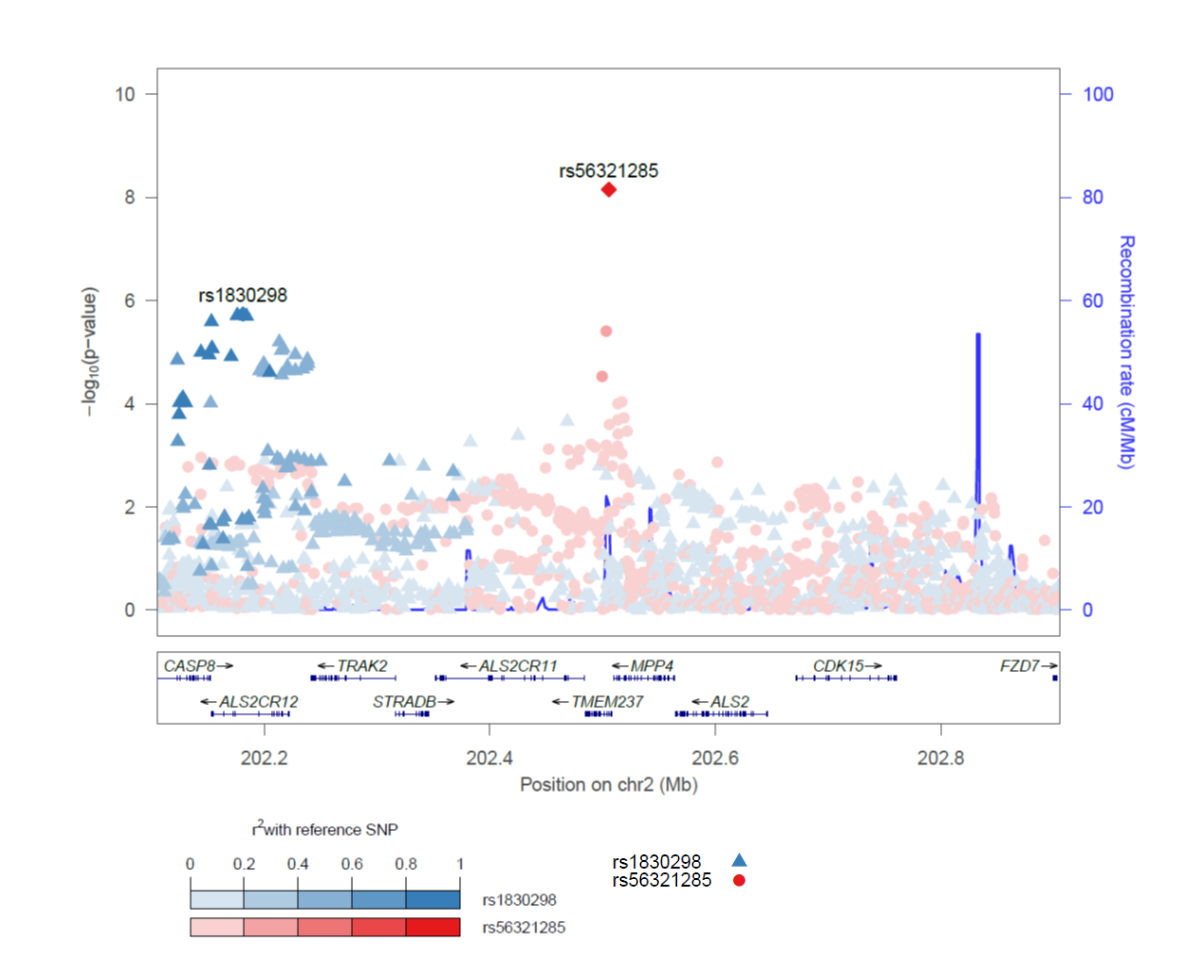

Supplement: S2 Fig — Chromosome positions (x-axis) and -log10 P-value (y-axis) SqCC meta-analysis at 2q33.1. Genetic variants colored red according to their LD with rs56321285 (2q33.1 lead SNP) and colored in blue according to LD values with second lead SNP rs1830298. rs563321285 and rs1830298 r2 = 0.02. (TIF) [file pgen.1009254.s002.tif]

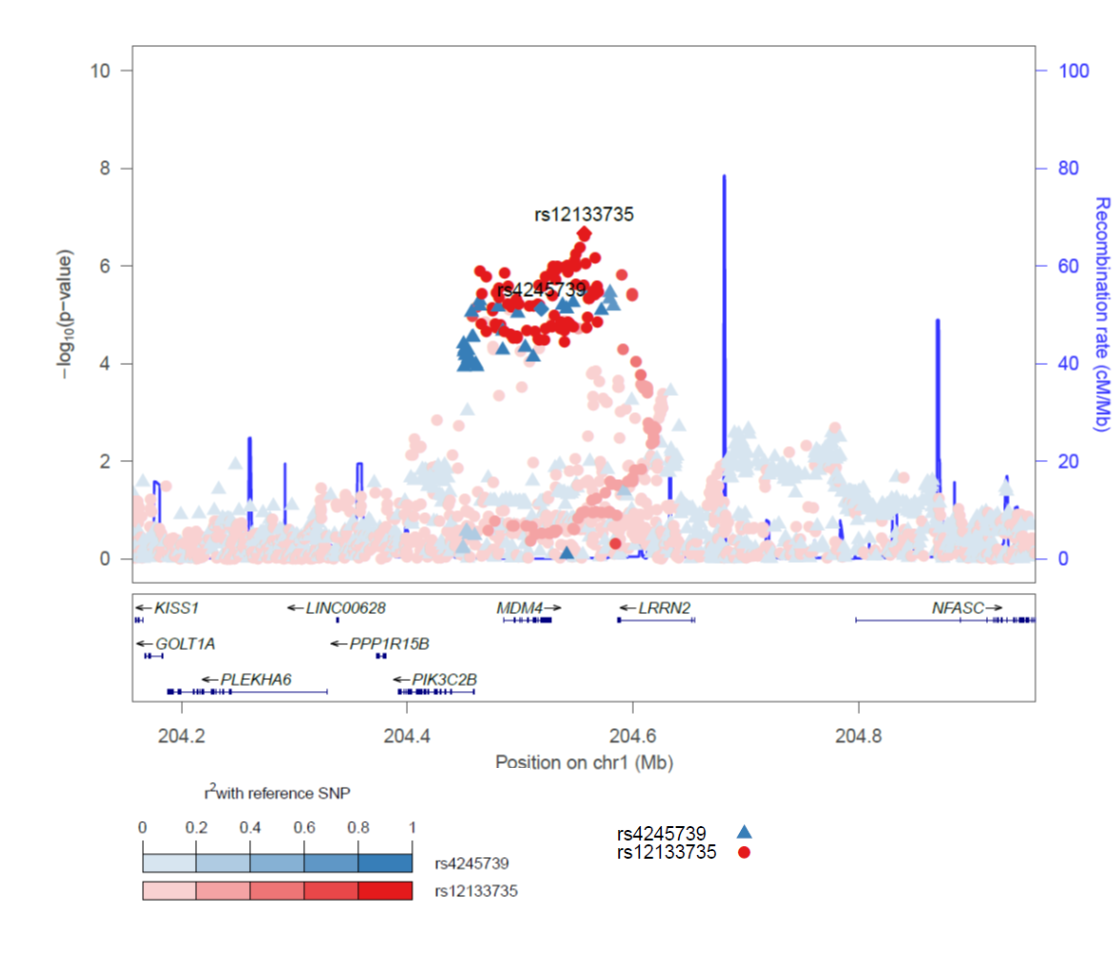

Supplement: S3 Fig — Chromosome positions (x-axis) and -log10 P-value (y-axis) of SqCC F-E meta-analysis at 1q32.1. Genetic variants are colored according to their LD with the rs12133735 (red) and with rs4245739 (blue) a variant previously associated with cancer risk; rs12133735 and rs4245739 (r2 = 0.63). (TIF) [file pgen.1009254.s003.tif]

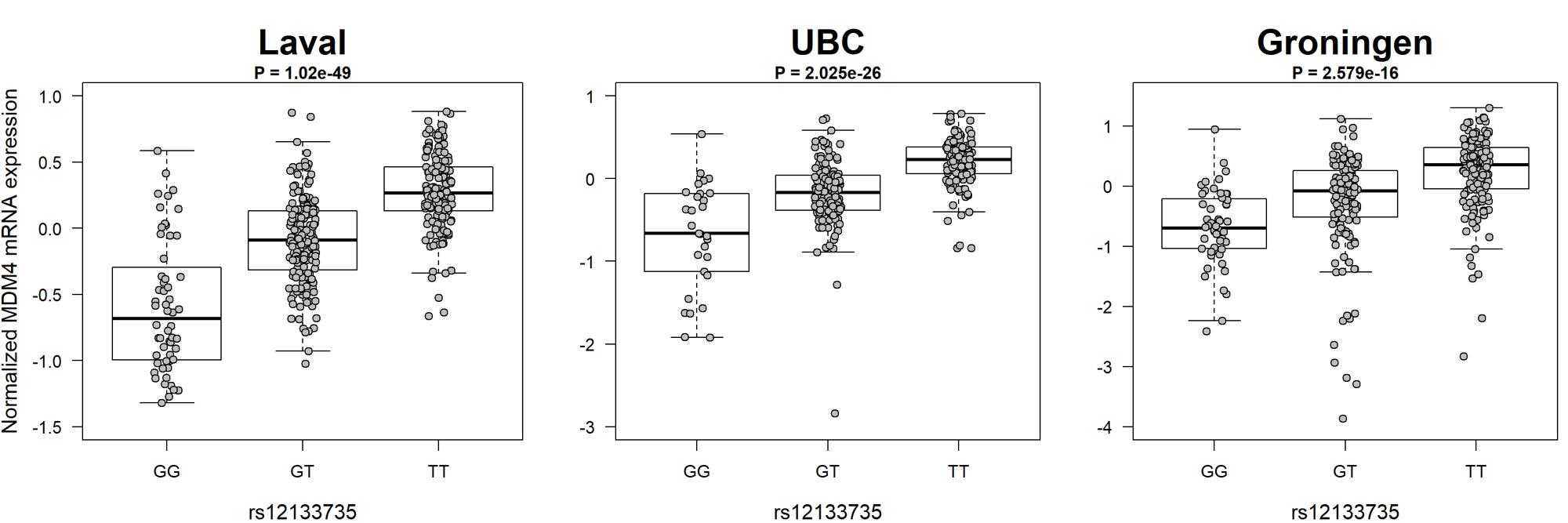

Supplement: S4 Fig — Boxplots for rs12133735 and MDM4 gene expression in 3 datasets from the Microarray eQTL study, from left to right: Laval University, University of British Columbia (UBC) and 3. University of Groningen. (TIF) [file pgen.1009254.s004.tif]

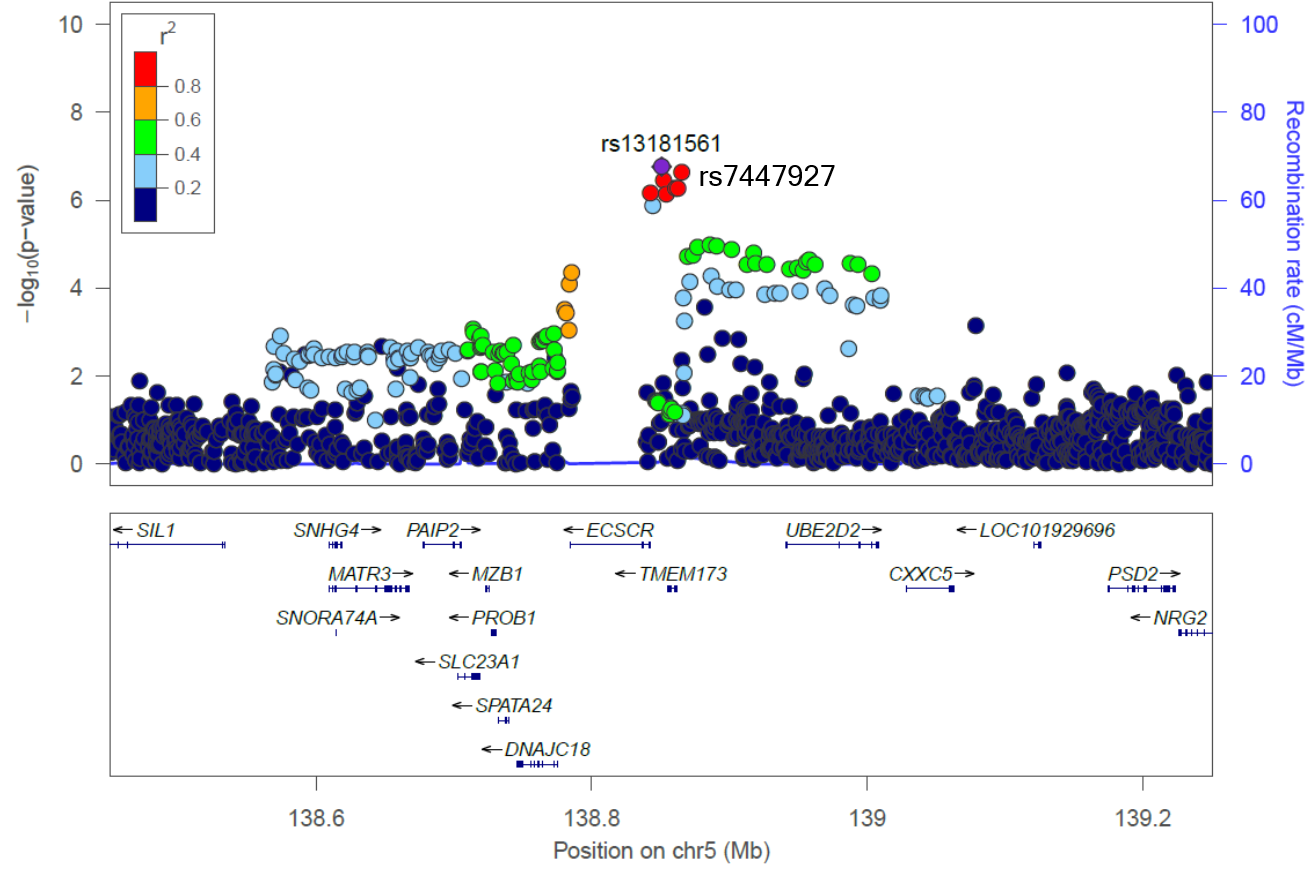

Supplement: S5 Fig — Chromosome positions (x-axis) and -log10 P-value (y-axis) SqCC F-E meta-analysis at 5q31.2. Genetic variants colored according to their LD with the labeled SNP (purple diamond). rs13181561 and rs7447927 (r2 = 0.94). (TIF) [file pgen.1009254.s005.tif]

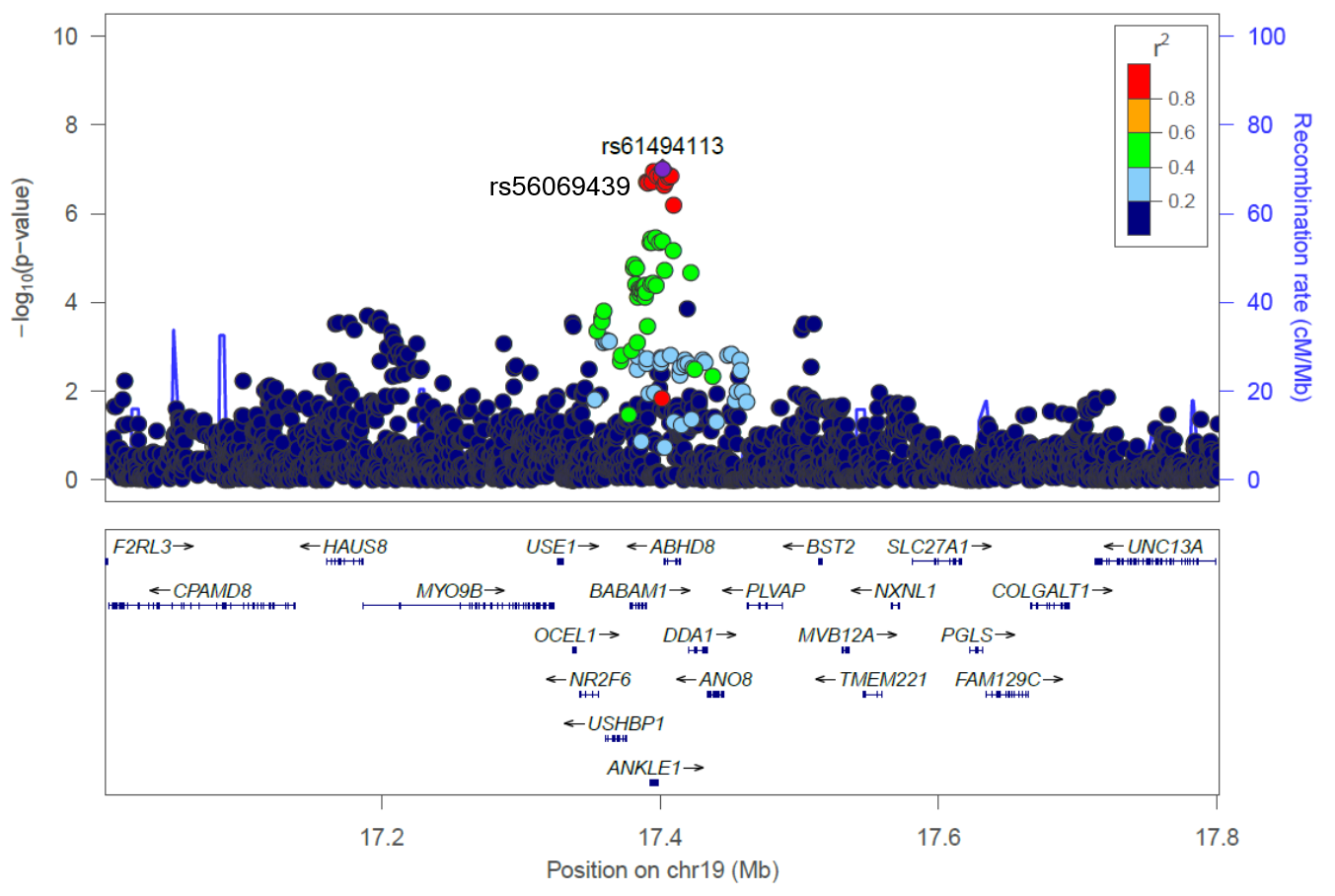

Supplement: S6 Fig — Chromosome positions (x-axis) and -log10 P-value (y-axis) SqCC F-E meta-analysis at 19p13.11. Genotyped and imputed variants colored according to their LD with the labeled SNP (purple diamond). rs61494113 and rs56069439 r2 = 1. (TIF) [file pgen.1009254.s006.tif]

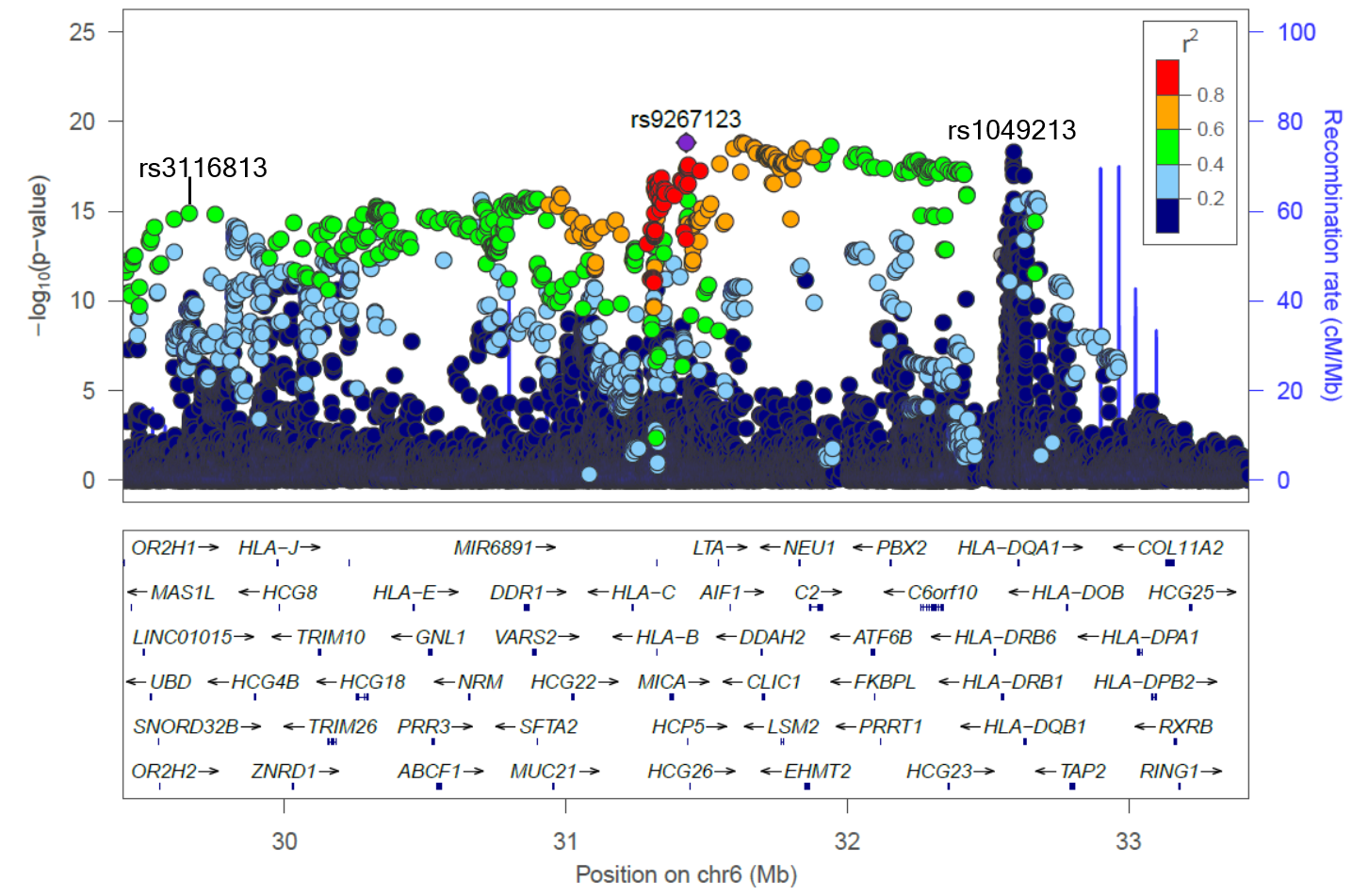

Supplement: S7 Fig — Chromosome positions (x-axis) and -log10 P-value (y-axis) SqCC F-E meta-analysis at 6p22.1- 6p21.33. Variants colored according to their LD with SNP rs9267123 (lead variant at 6p21.33). rs3116813 (6p22.1) is in moderate LD with rs9267123 (r2 = 0.5). rs1049213 at 6p21.33 is not correlated with rs9267123 (r2 = 0.01). (TIF) [file pgen.1009254.s007.tif]

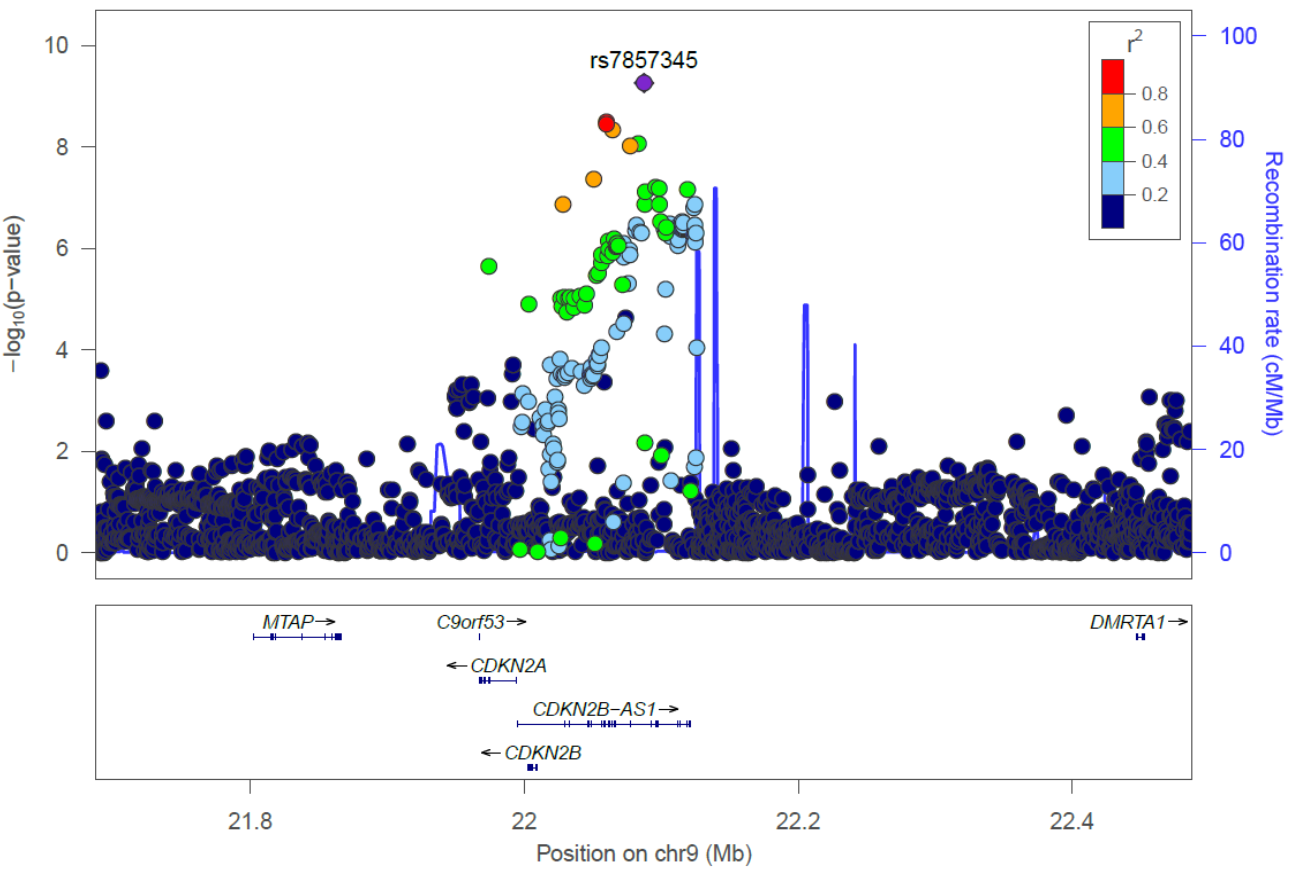

Supplement: S8 Fig — Chromosome positions (x-axis) and -log10 P-value (y-axis) SqCC meta-analysis at 9p21.3. Variants colored according to their LD with SNP rs7857345 (9p21.3 lead variant). (TIF) [file pgen.1009254.s008.tif]

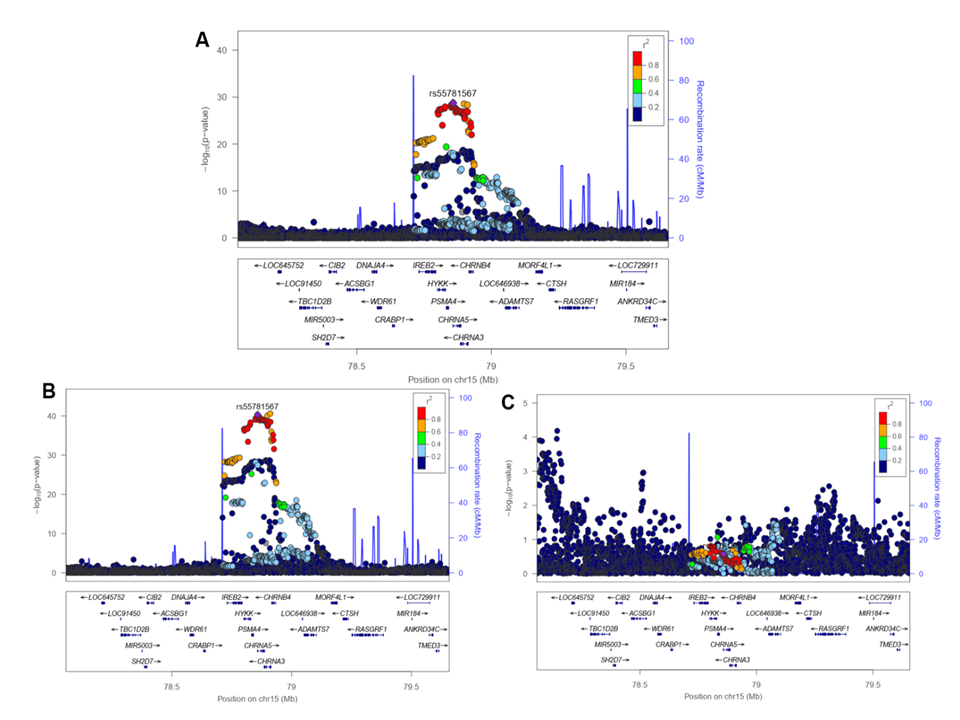

Supplement: S9 Fig — Regional association plot at 15q25 Chromosome positions (x-axis) and -log10 P-value (y-axis). A. aerodigestive SqCC P-values; B. Lung SqCC P-values; C. Oral and oropharyngeal cancer SqCC P-values. Genetic variants colored red according to their LD with rs55781567 (lowest P-value at 15q5 in the meta-analysis). (TIF) [file pgen.1009254.s009.tif]
