# Supplementary material for: Genome-wide association meta-analysis identifies pleiotropic risk loci for aerodigestive squamous cell cancers
Source: PLoS Genet. 2021 Mar 5;17(3):e1009254. doi: 10.1371/journal.pgen.1009254 (PMC7968735; doi:10.1371/journal.pgen.1009254)
Supplement: Attachment — Submitted filename: PlosGen_Review_Response_2.docx [file pgen.1009254.s021.docx]

Dear Drs. Chanock and McKinnon,

Re: PGENETICS-D-20-00903. “Genome-wide association meta-analysis identifies pleiotropic risk loci for aerodigestive squamous cell cancers”

Thank you for the opportunity to revise and resubmit for your consideration the above manuscript. We have provided point by point responses to the reviewers’ comments, and have revised the manuscript accordingly. We look forward to hearing from you.

Paul Brennan, PhD

**Reviewer #1**: This manuscript presents a meta-analysis of four aerodigestive squamous cell carcinoma (SqCC) GWAS. The study primarily searches for new loci that reach significance via meta-analysis and for evidence of pleiotropy at previously-identified loci (i.e., effects on two or more cancer subtypes). While power is limited, the analysis looks generally well-done. The authors find one new genome-wide significant locus (TMEM237) and three new loci with suggestive significance (P<5e-7); of the three suggestive loci, the MDM4 locus seems quite plausible. However, I have a concern about statistical significance in the pleiotropy analysis and have a few other questions and comments.

1. In their pleiotropy analyses, the authors used a significance threshold of P<5e-3 in at least one other cancer site, but they do not provide justification for this threshold. I am concerned that P<5e-3 does not sufficiently correct for multiple hypotheses tested, which at least include 10 loci x 3 other cancer sites. Also, was only one SNP tested per locus in these pleiotropy analyses, or were multiple SNPs tested? Line 343 "The lead SNP exhibiting pleiotropy..." suggests the latter; if so, then even more hypotheses were tested.

We thank the reviewer for this comment and have modified our approach to take these concerns into account. In particular, we now characterize a locus as pleiotropic using the following criteria: 1) SCC_meta_ P< 5x10^-7^ ; 2) at least two tumor sites with P<0.0001, and 3) same direction effect for all tumor sites. The p-values are from the SCC meta-analysis p-values and the summary association statistics for each cancer site (results: line 215, page 10 & methods: line 454 page 23).

2. Table 2 is titled "Novel genomic regions..." but only the first of the four listed associations is actually genome-wide significant. The authors should separate significant vs. suggestive results in the table. They could also consider moving some of the main text describing suggestive results (which is quite long) to a supplementary note.

We have added subtitles to Table 2 indicating genome-wide significant and suggestive results (page 13). We also reduced the text for the suggestive loci results.

3. The overview of analyses indicates that the pleiotropy analyses considered loci that previously reached P<5e-7 in a single-cancer analysis, but the 9p21.3 locus in Table 3 does not appear to satisfy this criterion. Perhaps they should just use a criterion of loci reaching genome-wide significance in their meta-analysis?

As described above we modified the approach to characterize loci as pleiotropic. With regards to rs7857345 at 9p21.3, was classified a pleiotropic because the SCC_meta_ P-value was <5x10^-7^, with another tumor site at P<5x10^-3^. We modified the language (in the results and the methods) to clearly specify the criteria used to classify loci as pleiotropic. Using the new criteria 9p21.3 is still a pleiotropic locus; SCC_meta_ P<5x10^-7^, the LuSqCC and OSqCC P < 5x10^-4^ and all the ORs in the same direction.

4. The authors note that their lead SNP at the MDM4 locus is an eQTL for MDM4 and that MDM4 upregulation can inactivate p53, leading to cancer. Is the eQTL effect direction consistent with this proposed mechanism?

The canonical function of MDM4 in normal cells is to bind to the N-terminal domain of p53 and inhibit transcriptional activity. MDM4 upregulation has been described in multiple tumors as an oncogenic mechanism to repress p53 {Haupt, 2019 #133}. In our study rs12133735-G (1q32.1) increases the risk of SqCC and was associated with lower MDM4 gene expression in normal lung tissues from the microarray study MDM4 eQTL, which in tumors would result in promotion of p53 activity and tumor suppression. Thus, the effect direction of the eQTL is not consistent with the detected association. However, these eQTL analyses were performed in normal lung tissue samples; MDM4 protein levels in normal tissues are generally low and do not correlated well with expression. Additionally, the MDM4 gene is subject to alternative splicing events; the two main MDM4 isoforms are the full-length transcript (MDM4-FL) and MDM4-Short (MDM4-S, that lacks exon 6). Proteins from these transcripts differ in their oncogenic potential and stability {Haupt, 2019 #133}. We thank the reviewer for this comment, and have now expanded the possible 1q32 SNP mechanism section to include these caveats.

5. A few software packages mentioned in the main text (e.g., ASSET and FUMA) are not defined until Methods. Providing brief descriptions in the main text would improve readability.

We added comments about these methods in the results section.

6. The legend of Figure 1 indicates that the y-axis was truncated at P=5e-30. Indicating the most significant P-value would be helpful (either in the figure or just by stating it in the legend).

We modified Figure 1 legend to include all the meta-analyses p-values.

**Reviewer #2:** This paper combines the results from genome-wide association studies (GWAS) of four aerodigestive squamous cell cancers in an effort to identify (a) novel loci that area associated with risk for more than one of the four cancers and (b) identify regions (perhaps already identified by one or more GWAS) that are plausibly associated with more than one cancer. The authors adopt two sensible and complementary approaches to combine results (i) a simple fixed-effect meta analysis (which will have greatest power when an alleles is associated with all four cancers in the same direction and magnitide) and (ii) a subset-based test (which allows for the possibility that not all cancers will be associated with an allele or in the same direction). They report one new locus and a handful of pleiotropic loci.

The paper's methods are reasonable and sound, and the results are reported with care and appropriate caution (given the quite small sample sizes for two of the studied cancers). Like many GWAS, the ultimate biological implications of the findings are unclear and left for others to sort out--not that there's anything wrong with that. My one substantive comment has to do with the striking lack of detectable pleiotropy at 15q25.1. Considering that lung cancer and head and neck cancer both have very strong genetic correlations with smoking behaviors (see ref 14), it's quite puzzling that the locus with the strongest cigarettes per day association is associated with LuSqCC but not OSqCC. This really deserves more comment--why do the authors think this is the case? Are the analyses from ref 14 not broken out by subtype, or at least not the same way as here?

We thank the reviewer for this important comment. Jiang et al reported a significant genome-wide genetic correlation between head and neck cancer (HNC) and lung cancer (r_g_=0.57), and between these cancers and smoking status (HNC-smoking status: r_g_=0.47; lung cancer-smoking status r_g_=0.56). In that study, they also used summary statistics from European individuals in the 2016 oral & oropharyngeal cancer GWA (referred as HNC) and the overall lung cancer (adenocarcinoma and squamous) GWA meta-analyses. Sub-type analyses were not conducted for HNC or lung cancer. For the smoking status and cancer genetic correlation analyses they used GWAS summary statistics from the UK biobank, and not smoking information from the cancer studies. Genome-wide genetic correlation is based on aggregated information across all SNPs in the genome; thus, it is possible that two traits with a significant genome-wide correlation (i.e. HNC & smoking status) have local genetic regions with low or no correlation. Additionally, Jiang et al also estimated local genetic correlations and found no significant associations at 15q25.1 between HNC and lung cancer (Supplementary data 2 ref).

In our study, we used a meta-analysis approach to evaluate relations between aerodigestive squamous cancers (SqCC). As mentioned in the results, 15q25 was prominent (P<5x10^-8^) in the SqCC meta-analyses mainly due to the contribution of lung SqCC but not of OSqCC. **Figure 1** shows local association plots at the 15q25 for the aerodigestive SqCC meta-analyses (A), lung SqCC (B) and OSqCC (C). Given the strong associations between 15q25 and lung cancer and smoking behavior, it would be expected to see a signal in this area for another smoking linked neoplasia such as oral/oropharyngeal squamous cancers (OSqCC). However, based on the results of this study and all other published HNC GWAS (including those of others) 15q25 variants have not been previously been identified at P<5 x10^-8^ or P<5 x10^-7^ (**Table 1**). In our previous study, we reported 2 nearby associations at 15q21 and 15q26 (P<5e-7); however, these SNPs were not in LD with 15q25 variants. We incorporated these plots as a supplemental figure in the manuscript and expanded our commentary of this issue (line 335, page 18).

**Figure 1.** Regional association plot at 15q25 Chromosome positions (x-axis) and -log10 P-value (y-axis). A. aerodigestive SqCC P-values; B. Lung SqCC P-values; C. Oral and oropharyngeal cancer SqCC P-values. Genetic variants colored red according to their LD with rs55781567 (lowest P-value at 15q5 in the meta-analysis).

**Table 1**. Loci with reported HNC associations (P<5x10^-7^) in previous GWA studies

| **Study** | **Phenotype** | **Ancestry** | **Regions** |
| --- | --- | --- | --- |
| [21437268](http://www.ncbi.nlm.nih.gov/pubmed/21437268)  McKay et al 2011 | Oral cavity & pharyngeal cancer | European | 4q23; 4q21.23; 12q24.13 |
| [25129146](http://www.ncbi.nlm.nih.gov/pubmed/25129146)  Wu et al 2014 | Esophageal squamous cell carcinoma | Chinese | 2q33.1; 5q11.2; 5q31.2; 6p21.32; 8q24.22; 9p21.3; 10q23.33; 17p13.1; 21q22.12 & 21q22.12 |
| [25194280](http://www.ncbi.nlm.nih.gov/pubmed/25194280)  Wei et al 2014 | Laryngeal squamous cell carcinoma | Chinese | 5q14.3; 6q16.1; 6p21.33; 11q12.2; 12q24.21; 16p13.2 |
| [27749845](http://www.ncbi.nlm.nih.gov/pubmed/27749845)  Lesseur et al 2016 | Oral cavity & pharyngeal cancer | European/admix | 4q23; 5p14.3; 6p21.32; 9q34.12; 11p15.4 |
| [27749845](http://www.ncbi.nlm.nih.gov/pubmed/27749845)  Lesseur et al 2016 | Oral cavity cancer | European/admix | 2p23; 4q23; 5p15.33; 6p21.32; 6p21.33; 9p21.3; 9q34.12; **15q21.2; 15q26.2** |
| [27749845](http://www.ncbi.nlm.nih.gov/pubmed/27749845)  Lesseur et al 2016 | Oropharynx cancer | European/admix | 4q23; 6p21.33; 6p21.32 |
| [32276964](http://www.ncbi.nlm.nih.gov/pubmed/32276964)  Shete et al 2020 | Head & neck squamous cell carcinoma | European | 6p22.1; 6p21.32; 6p21.32 |
| [32276964](http://www.ncbi.nlm.nih.gov/pubmed/32276964)  Shete et al 2020 | Oral cavity cancer | European | 5p15.33; 6p21.32 |
| [32276964](http://www.ncbi.nlm.nih.gov/pubmed/32276964)  Shete et al 2020 | Oropharynx cancer | European | 2p23.1; 6p21.33; 6p21.32 |
| [32276964](http://www.ncbi.nlm.nih.gov/pubmed/32276964)  Shete et al 2020 | Hypopharyngeal or laryngeal cancer | European | 10p15.1; 18q22.2 |

My remaining comments are minor presentation and grammar points. One more good read over by a copyeditor to clean up run-on sentences, subject-verb agreement and the like would be helpful.

line 217 "...fixed-effects (F-E) ^{meta-analysis} and a subset-based meta-analysis ^{using ASSET}." To disambiguate.

This has been corrected.

lines 220-221 "Loci that reached suggestive .... considered noteworthy." Circular definitions. "Colors we defined as red were labeled as crimson." Instead: "Loci with p<5e-7 were considered noteworthy" or "We defined suggestive genome-wide significance as p<5e-7."

We have corrected these issues.

line 225 Can you give some motivation as to why p<5e-3 was considered as evidence for pleiotropic effects on a trait at a locus known to be genome-wide significantly associated with another trait?

As described above, we have now re-defined the criteria for pleiotropy of known SqCC loci: 1) SCC_meta_ P< 5x10^-7^ ; 2) At least two tumor sites with P<5x10^-4^, and 3) Same direction effect for all tumor sites.

lines 226-228. "Additionally... 19p13.11." This is not a sentence. Delete "that"

We modified this sentence (page 216 line 10).

lines 273-276. Not a sentence. Seems to be two sentences pasted together, with the second sentence missing a verb.

We modified this sentence

line 306 "this analysis" not "this analyses"

We modified throughout the manuscript.

line 333 state the reference panel used to calculate r2. Obviously the r2 cannot be 1 in the study samples or the p-values for rs614etc and rs560etc would be identical.

We included the reference panel in all r2 in the results.

lines 344-346 and throughout the text (tables are better about this): SNPs are not associated with increases or decreases in risk, alleles (relative to other alleles) are. If you must use "[xxx] is associated with increased risk" language, you have to refer to the effect allele. "rs12345[G] is associated with an increased risk," not "rs12345 is associated with an increased risk."

line 352 see comment on 344-346.

line 358 see comment on 344-346.

line 365 see comment on 344-346.

We modified all the results to include the SNP and the allele.

line 430 no comma after based

Comma added (line 360, page 19)

lines 442-443 I don't know how "interesting" this observation is. You see what you are powered to see. There may be lots of pleiotropic loci with heterogeneous effects, but in these sample sizes, they may be hard to detect--certainly less power than the SNPs where everything aligns. This sentence is a little tautological, a little post-hoc-power-calculation-y--"we were well powered to detect the things we detected." You should note that there may be heterogeneous pleiotropic loci that you've missed b/c of low power.

We removed the word “interestingly” and added the commentary to acknowledge for the possibility of heterogenous associations (line 380, page 20)

line 455 discovered not discover

This has been corrected (line 393 page 20)

lines 457-459 Run on. Semi-colon or full stop after "limited."

Added semicolon.

**Reviewer #3**: This manuscript describes an analysis in which GWAS results from multiple from multiple aero-digestive squamous cell cancers (lung, oral cavity, oropharynx, larynx, and esophagus) are combined in order to identify loci with pleiotropic effects on multiple cancer types. This is a novel and unexplored hypothesis. There is one novel association observed at a genome-wide significance P-value threshold (in the TMEM237 gene region). All other loci passing this threshold have been reported previously for specific cancer types. The authors report several suggestive association signals and describe pleiotropy for previously identified association signals.

My primary concerns is the lack of replication for the novel locus and the strong emphasis on describing and characterizing the suggestive association signals (which seems like more attention that is warranted based on the statistical evidence). A few additional comments are below:

We thank the reviewer for this comment. An independent replication would be the ideal approach to establish these loci as susceptibility variants for aerodigestive SqCC. However, this meta-analysis includes the largest studies of these cancer types available in populations of European ancestry, and it is thus not feasible to find a similar large study for an independent replication of these findings. We have stated this as a limitation of our study in the manuscript. Although not replicated, these observations grant biological plausibility to our findings. We have also added a note of caution for our results with at borderline GWA significant P-value (line 397, page 21).

Introduction: On point of clarification: this GWAS is Europeans only? Or does it also include individuals of European ancestry from outside of Europe?

We clarified (abstract: line 122 page 6; overview section of the results: line 198, page 9) that this study included individuals of European ancestry. In the methods, we specify that this analysis included individuals from multiple epidemiological studies in Europe, North and South America (line 418, page 22).

In my view, there is an over-emphasis of “suggestive” association signals in this paper (i.e., associations with P<10-7). Several signals passing this threshold are expected under the null. A substantial amount of text is this paper is devoted to discussing the biological and epidemiological evidence supporting regions identified at this suggestive P-value threshold.

We have shortened the results on the suggestive association signals. We have also added a note of caution that some of our results with a borderline GWA significant P-value (on both sides of P=5x10-8) will benefit from further future replication (line 397, page 21).

A P-value threshold of 5x10-3 was used to identify pleiotropic loci. How was this threshold determined? Consider a systematic approach to test for pleiotropy with an explicit multiple testing adjustment.

As discussed in response to reviewer 1, we have not applied a more stringent criteria for the pleiotropy analyses (results: line 215, page 10 & methods: line 454 page 23).
