# Supplementary material for: Genome-wide association meta-analysis identifies pleiotropic risk loci for aerodigestive squamous cell cancers
Source: PLoS Genet. 2021 Mar 5;17(3):e1009254. doi: 10.1371/journal.pgen.1009254 (PMC7968735; doi:10.1371/journal.pgen.1009254)
Supplement: Attachment — Submitted filename: PlosGen_Review_ResponseV2-2Nov20.docx [file pgen.1009254.s022.docx]

Dear Drs. Chanock and McKinnon,

Re: PGENETICS-D-20-00903R1. “Genome-wide association meta-analysis identifies pleiotropic risk loci for aerodigestive squamous cell cancers”

Thank you for the opportunity to revise and resubmit the above manuscript. Below, we provide point by point responses to the remaining reviewers’ comments, and revised the manuscript accordingly. We look forward to hearing from you.

Sincerely,

Paul Brennan, PhD

*Comments to the Authors:*

*Reviewer #1: The authors have satisfactorily addressed my comments. Providing some justification for the significance criterion of "two single cancer association results at P<5x10-4" would still be helpful. (The response letter seems to have a typo and states P<0.0001 instead of P<5x10-4.)*

We thank the reviewer for this comment. In an effort to address this concern from your previous comments, we set a more stringent criteria to classify loci as pleiotropic; we listed these criteria in the results overview as well as in the methods (results: line 215, page 10 & methods: line 454 page 23). Briefly, we now characterize a locus as pleiotropic using the following criteria: 1) SCCmeta P< 5x10-7 ; 2) at least two tumor sites with P<5x10-4, and 3) same direction effect for all tumor sites. The p-values are from the SCC meta-analysis p-values and the summary association statistics for each cancer site.

We recognize that these criteria are arbitrary, but we tried to be stringent while also accommodating for the sample size imbalances in our datasets. We have now added a statement in the limitations of our study acknowledging this (discussion: line 400, page 21).

*Reviewer #2: The authors have thoughtfully responded to my comments.*

*Reviewer #3: The authors have addressed my comments.*

*Have all data underlying the figures and results presented in the manuscript been provided?*

*Large-scale datasets should be made available via a public repository as described in the PLOS Genetics data availability policy, and numerical data that underlies graphs or summary statistics should be provided in spreadsheet form as supporting information.*

*Reviewer #1: Yes*

*Reviewer #2: No: It is not clear that summary statistics for individual cancers or the cross-cancer analyses have been made publicly available. Hence it would be nearly impossible to reproduce the Manhattan plot--researchers would have to download dbGAP data sets, run their own QC and GWAS analyses, then run their own cross-cancer analyses. Inevitably, small differences in analysis pipelines will lead to different results.*

We added a new supplemental table included all meta-analyses summary statistics for all SNPs with *P*<5x10^-5^ for Figure 1 Manhattan plot (Table S2). The summary statistics for the lung squamous dataset are deposited in dbGaP (phs001273.v3.p2). The oropharyngeal GWAS summary statistics are in the process of being submitted to the MR BASE and IEU Open GWAS platforms (<https://gwas.mrcieu.ac.uk/>). We added comments stating this in the methods.

*Reviewer #3: Yes*
